# Alfalfa polysaccharide prevents H_2_O_2_-induced oxidative damage in MEFs by activating MAPK/Nrf2 signaling pathways and suppressing NF-κB signaling pathways

**DOI:** 10.1038/s41598-018-38466-7

**Published:** 2019-02-11

**Authors:** Lixue Wang, Yuhuai Xie, Weiren Yang, Zaibin Yang, Shuzhen Jiang, Chongyu Zhang, Guiguo Zhang

**Affiliations:** College of Animal Science and Veterinary Medicine, Shandong Provincial Key Laboratory of Animal Biotechnology and Disease Control and Prevention, Shandong Agricultural University, Taian, Shandong China

## Abstract

Alfalfa polysaccharide (APS) is a bioactive component extracted from alfalfa that exhibits potent antioxidant properties. However, the cellular and molecular mechanisms underlying these properties remain unclear. To explore the molecular mechanism by which APS exerts antioxidant effects, an H_2_O_2_-induced oxidative stress mouse embryonic fibroblast (MEF) model was established. Cell proliferation, antioxidant enzyme activity, immune cytokine expression, and related protein expression were examined in APS-supplemented or non-supplemented conditions. The results suggested that APS strengthened the antioxidative capacity of MEFs, increasing cell proliferation, superoxide dismutase activity (SOD), and the total antioxidant capacity (T-AOC). In addition, APS reduced the secretion of interleukin (IL)-6 and IL-8 as well as expression of the proinflammatory gene retinoic acid-inducible gene I (RIG-I). APS was also able to activate the mitogen-activated protein kinase (MAPK) pathway, which promoted the translocation of nuclear factor erythroid 2–related factor 2 (Nrf2) to the nucleus. However, expression of nuclear factor-κB (NF-κB) was decreased after APS treatment. Overall, these results suggest that APS relieves H_2_O_2_-induced oxidative stress in MEFs by activating MAPK/Nrf2 signaling and suppressing NF-κB signaling. To the best of our knowledge, this is the first study to link APS with MAPK/Nrf2, NF-κB and RIG-I, thus providing new perspectives regarding the mechanisms of the antioxidant activity of APS.

## Introduction

Oxidative stress, which results from an imbalance between oxidants and reductants at the cellular or organismal level, plays an important role in the development of numerous types of diseases, such as pneumonia, enteritis, and sepsis^1^. The most well-investigated causes of oxidative stress in veterinary medicine are metabolic and inflammatory events and environmental factors (heat stress, malnutrition, and other factors). Most cells have evolved intricate mechanisms to prevent the generation of reactive oxygen species (ROS) or to detoxify ROS via the activation of antioxidant/detoxification enzymes, which enhance cellular ROS scavenging capacity to maintain cellular redox homeostasis and reduce oxidative damage; the balance between ROS production and antioxidant defense determines the degree of oxidative stress. Excess ROS can perturb the normal redox balance and lead to cellular oxidative stress, which has consequences that include modifications of cellular proteins, lipids and DNA. The most widely studied type of oxidative stress involving protein modification is the formation of carbonyl derivatives^2,3^. Malondialdehyde (MDA), the principal and best-studied product of polyunsaturated fatty acid peroxidation, is the product of lipid oxidation^4^. Oxidative stress and the accumulation of ROS/reactive nitrogen species (RNS) can lead to a number of different types of DNA damage, including direct modification of nucleotide bases, formation of apurinic/apyrimidinic sites, DNA single-strand breaks (SSBs), and, much less frequently, DNA double-strand breaks (DSBs).

A diverse range of chemicals (vitamins, microelements and synthetic antioxidants) have been reported to possess antioxidant activities^5–7^. However, some physical properties of synthetic antioxidants, such as their high volatility and instability at elevated temperatures, along with strict legislation of their production and use, their carcinogenic properties, and consumer preferences, have shifted the attention of manufacturers from synthetic to natural antioxidants^8^. In fact, various phytochemicals in moderate amounts exert antioxidative effects by enhancing cell viability and activating signaling pathways involving cell survival, antiapoptotic mechanisms and antioxidant defense^9–11^. Nonetheless, substantial evidence is needed to extensively elucidate the biomedical significance of these chemicals and the underlying mechanisms of their function.

Alfalfa, the most popular forage crop, is widely used in animal husbandry; it is a high-quality protein source for feed with advantages such as a high nutrient value, vigorous resistance and high yield. Alfalfa polysaccharide (APS) is one of the main bioactive components extracted from alfalfa, and several studies have shown that APS consists of glucose, mannose, rhamnose, and galactose^12^. These monosaccharide components are the pharmacophores of TLR4-related active polysaccharides^13^. Previous studies have proven that APS inhibits 1, 1 - diphenyl - 2 - picrylhydrazyl (DPPH) radicals and enhances growth performance and antioxidant status in heat stressed-rabbits^14^, and APS can also protect hepatocytes against oxidative injury^12^. In addition, hydroxyl radicals can easily induce oxidative damage by crossing cell membranes, and there is a positive correlation between the polysaccharide concentration and hydroxyl radical-scavenging activity^15^.

H_2_O_2_ is widely used as an inducer of cellular oxidative stress. The production of ROS can be increased by oxidative stress, and it has been reported that overall longevity is most strongly associated with ROS production^16^. Although the relevance of *in vitro* senescence to organismal aging is unknown, several studies have indicated that oxidants are important in the development of the senescence phenotype^3^. Mitochondrial DNA is more susceptible than nuclear DNA to the oxidative damage involved in senescence^17^. Therefore, cell senescence can be regarded as an aging index, and it has been reported that mouse embryonic fibroblasts (MEFs) are a vital senescence-like model^18^.

Previous studies have found that alfalfa can protect against oxidative damage in fatty liver disease and hepatocyte injury^12,19^, although the underlying mechanisms remain unclear. We speculate that the antioxidant activity of APS might be attributable to a reduction in oxidative damage. To investigate this possibility, an *in vitro* MEF model of oxidative stress induced by H_2_O_2_ was established. We then characterized the antioxidative activities of APS in these cells and examined the signaling pathways involved.

## Results

### Construction of an H_2_O_2_-induced oxidative stress model in MEFs

**Figure 1:**
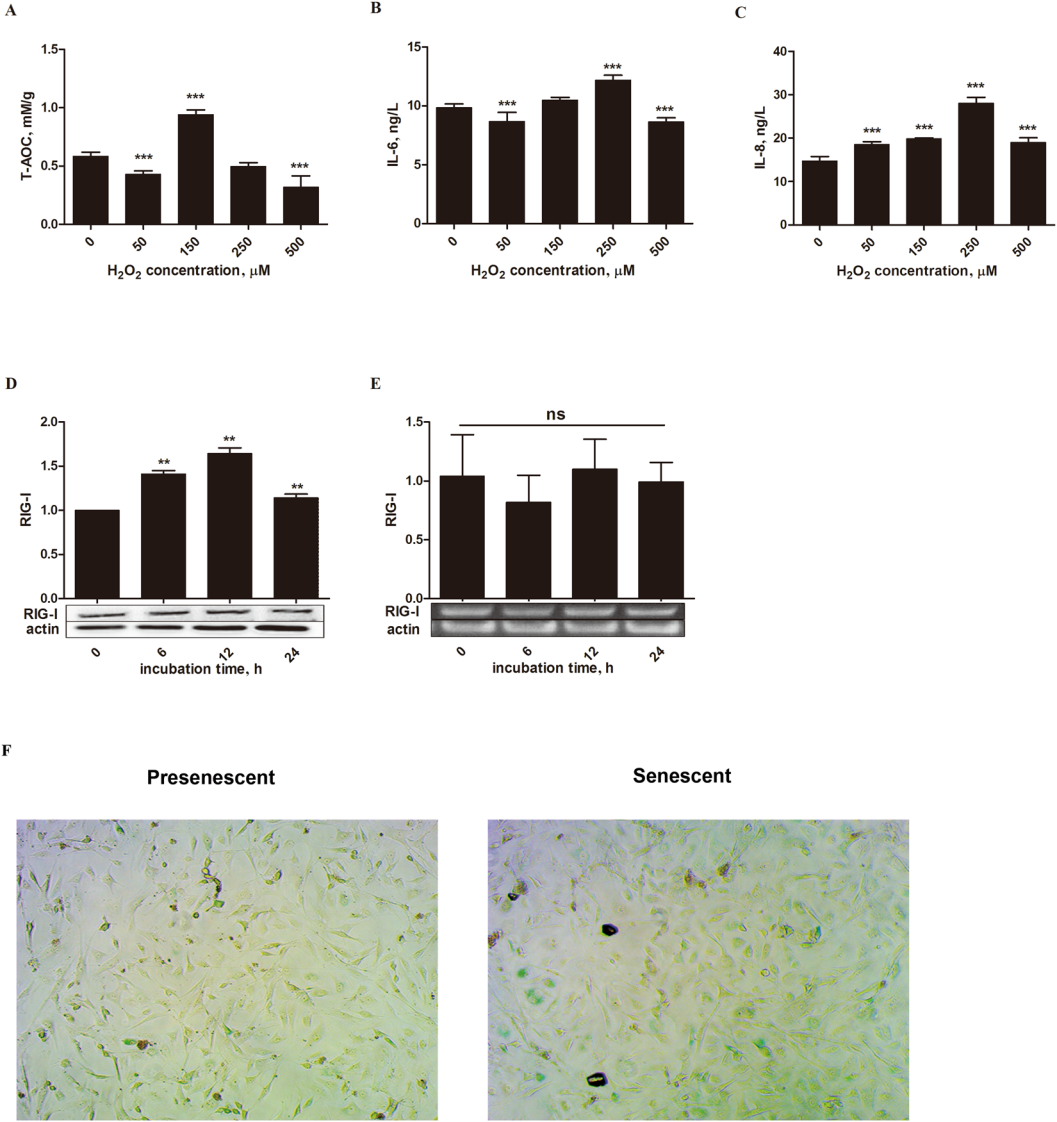
Construction of the H_2_O_2_- induced MEFs oxidative stress model. Cells were treated with various concentrations (0, 50, 150, 250, and 500 µM) of H_2_O_2_ for 12 h, and then T-AOC, IL-6 and IL-8 levels were determined (**A**–**C**). Cells were also stimulated for different times (0, 6, 12, and 24 h) with 250 µM H_2_O_2_ (**D**,**E**). The protein levels and gene expression of RIG-I were detected by Western blotting and q-RT PCR, respectively. The band intensities were analyzed using image analysis software. (**F**) H_2_O_2_ (250 µM) was used to stimulate cells for 12 h, and the cells were then visualized with an InCell Analyzer 2000 (20X objective). The data are presented as the mean ± SD (n = 3 per group), and the significance relative to the control group was determined using Duncan’s multiple-range test. **P < 0.01 and ***P < 0.001; ns, no significant difference.

To demonstrate the antioxidant function of APS, we first constructed oxidative stress models with H_2_O_2_ in MEFs. Then, the cells were incubated with APS to decrease oxidative damage. MEFs are often used to research senescence, which is a form of oxidative damage^18,20^. First, cells were stimulated for 12 h with different concentrations of H_2_O_2_ (0, 50, 150, 250 and 500 µM). Upon stimulation with 150 µM H_2_O_2_, the total antioxidant capacity (T-AOC) was close to 1.0 mM/g, which was higher (*P* < 0.001) than the level in the other concentration groups (Fig. 1A). However, the activity of the inflammatory factor IL-6 and the chemokine IL-8 reached the maximum level (*P* < 0.001) when 250 µM H_2_O_2_ was added (Fig. 1B,C). These results revealed that when the cells were exposed to oxidative stress (150 µM H_2_O_2_), their antioxidant capacity was activated to prevent damage. With increasing H_2_O_2_, the oxidative damage exceeded the antioxidant capacity, resulting in decreasing T-AOC and increasing IL-6 and IL-8 levels. Inflammation damage and reduced antioxidant capacity ensued in this case because of oxidative stress. Therefore, 250 µM H_2_O_2_ may be the optimal concentration to stimulate oxidative stress in MEFs.

Next, cells were stimulated for 0, 6, 12, and 24 h with 250 µM H_2_O_2_ to test the effects of different durations of H_2_O_2_ stimulation. Retinoic acid-inducible gene I (RIG-I) is a proinflammatory gene that is correlated with senescence^21^. Although gene expression of the RIG-I transcript remained stable (*P* = 0.602), expression of the RIG-I protein was higher (*P* < 0.01) after stimulation with 250 μM H_2_O_2_ for 12 h than after other stimulation durations (Fig. 1D,E). Based on these four results, we found that the best way to construct the oxidative stress model was to stimulate cells with 250 μM H_2_O_2_ for 12 h.

Finally, after optimizing the conditions, we stained the cells to verify whether the cells were in senescence compared with the control. The cells were stimulated with 250 μM H_2_O_2_ for 12 h, and after staining, aging cells were colored green (Fig. 1F).

### Effect of APS treatment on H_2_O_2_-induced MEFs oxidative damage

**Figure 2:**
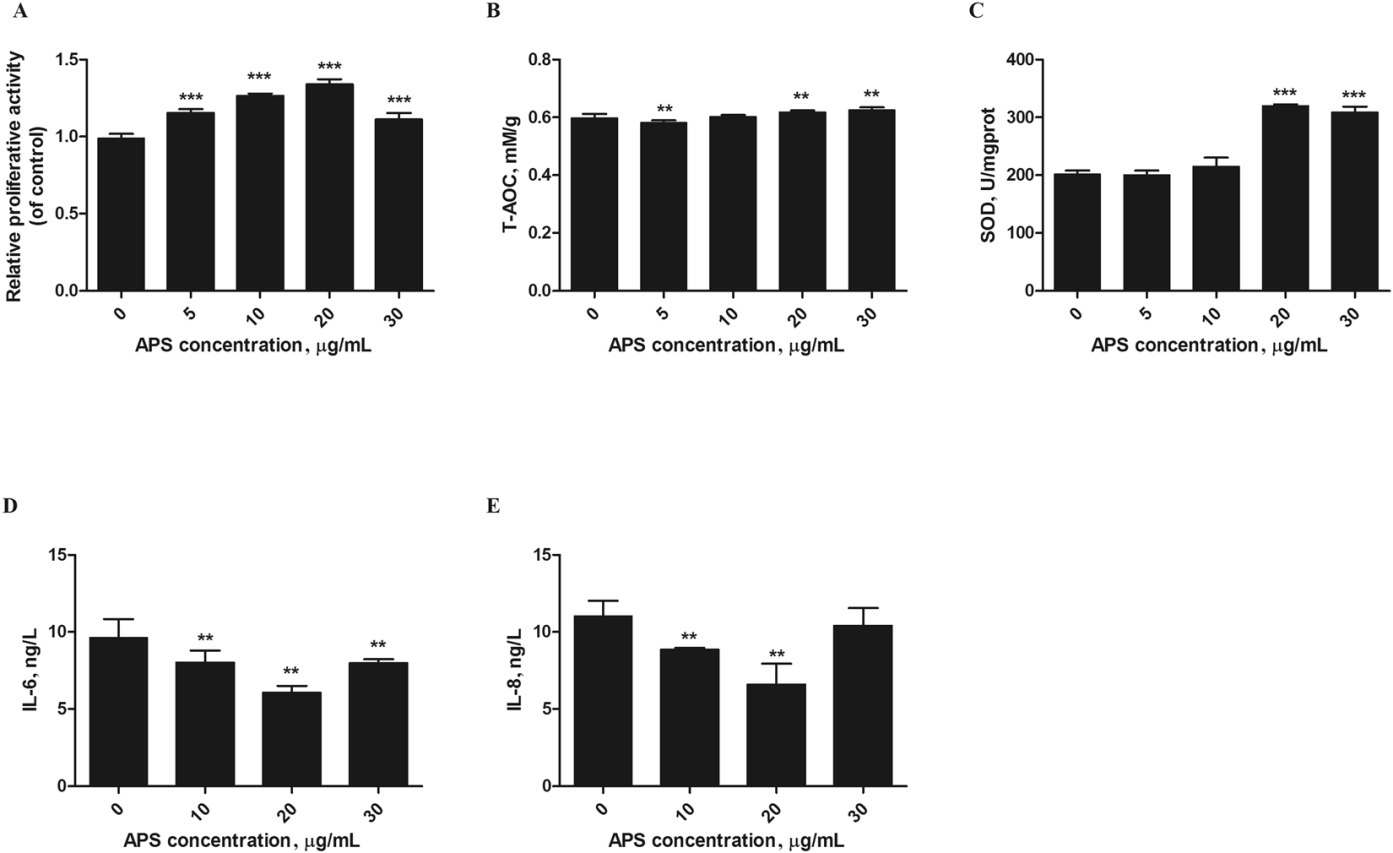
APS protected MEFs against H_2_O_2_-induced cell damage. MEFs were stimulated with 250 μM H_2_O_2_ for 12 h, and then incubated with different APS concentrations (0–30 µg/mL). (**A**) Cell proliferation was then measured using the MTT method. (**B**,**C**) The T-AOC and SOD activity in the cells were analyzed at 450 nm. (**D**,**E**) The concentrations of IL-6 and IL-8 were detected by ELISA. The values represent the mean ± SD (n = 3 per group); **P < 0.01 and ***P < 0.001.

To investigate the anti-inflammatory and antioxidative effects of APS, H_2_O_2_-induced MEFs were treated with APS at concentrations ranging from 0 to 30 μg/mL for 12 h. The cell proliferation was determined by MTT assay. As shown in Fig. 2A, the proliferative activity of H_2_O_2_-induced MEFs was enhanced after treatment with 0–20 μg/mL APS (*P* < 0.001). By contrast, cells treated with APS at a concentration of 30 μg/mL had lower proliferation than those treated with 20 μg/mL APS. This biphasic dose-response phenomenon is in agreement with the typical characteristics of *Glycyrrhiza glabra* extract^22^.

With the intensification of inflammation, damaged sites exhibit increased uptake of oxygen and thus increased release and accumulation of ROS^23^, and we hypothesized that APS could attenuate H_2_O_2_-induced cell damage. To test this hypothesis, cells were treated with 0–30 μg/mL APS for 12 h after being stimulated with 250 μM H_2_O_2_ for 12 h. As shown in Fig. 2, APS increased T-AOC and superoxide dismutase activity (SOD). In particular, 20 μg/mL APS exhibited the greatest (*P* < 0.01) antioxidative effect compared with H_2_O_2_ alone (Fig. 2B,C). In addition, we determined whether APS could protect cells against H_2_O_2_-induced inflammation. The results of ELISA showed that IL-6 and IL-8 secretion were significantly inhibited by APS (*P* < 0.01). At an APS concentration of 20 μg/mL, the IL-6 and IL-8 concentrations reached a minimum (Fig. 2D,E). The results demonstrated that APS exerted a protective action against H_2_O_2_-induced oxidative damage.

### APS upregulated TLR4/MyD88/IRAK1/TRAF6 production and reduced expression of NF-κB and RIG-I

**Figure 3:**
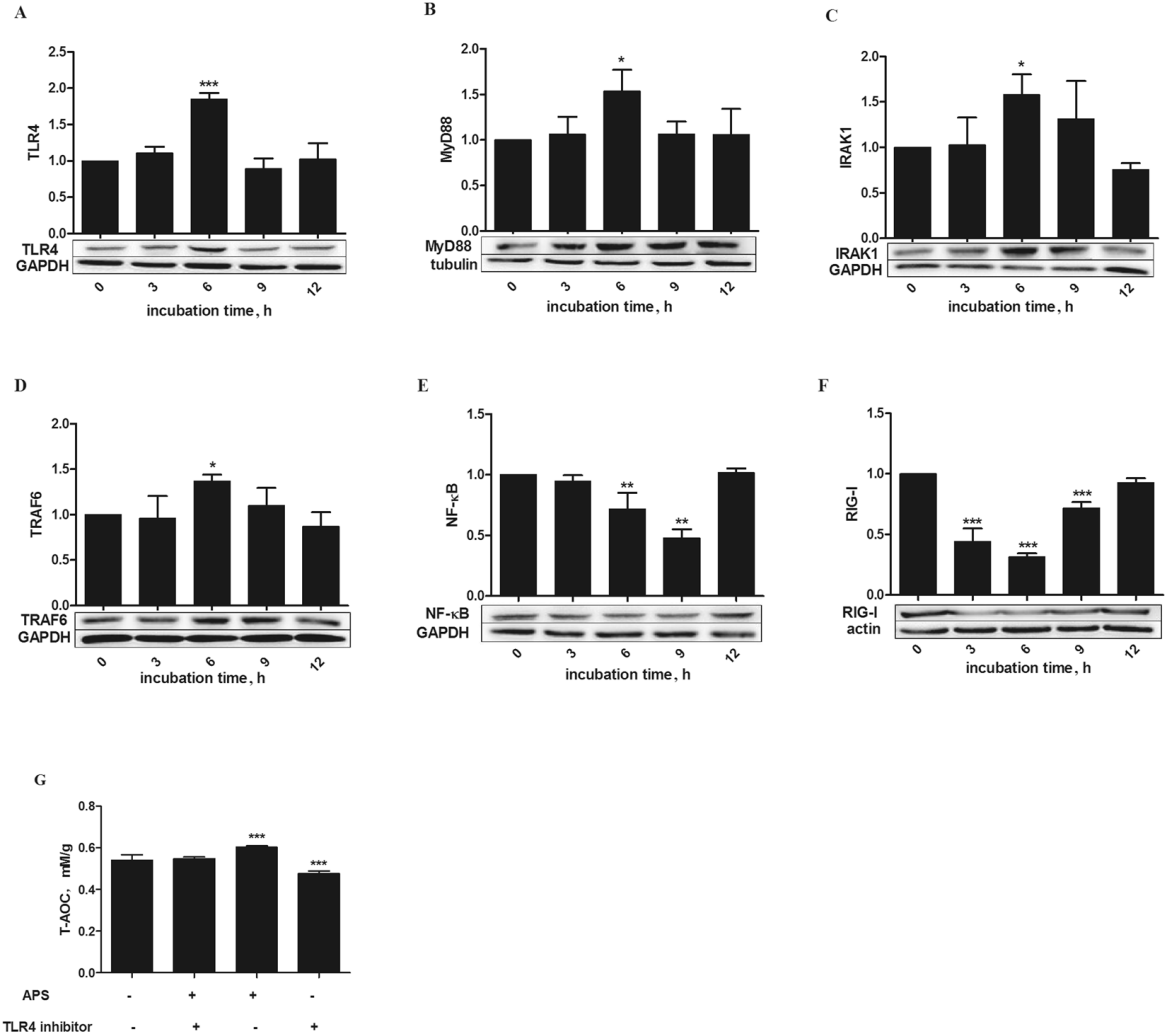
Effects of APS on expression of key proteins in the TLR4 signaling pathway, NF-κB and RIG-I in H_2_O_2_-pretreated MEFs. (**A**–**F**) Cells were treated for varying times (0, 3, 6, 9 and 12 h) with 20 µg/mL APS after stimulation with 250 µM H_2_O_2_ for 12 h. Proteins levels were determined by Western blotting, and the band intensities were analyzed using image analysis software. (**G**) Cells were stimulated with 250 µM H_2_O_2_ for 12 h. The MEFs were incubated in the presence or absence of a TLR4-neutralizing antibody (20 μg/mL) for 1 h and then treated with 20 μg/mL APS for 6 h, and the T-AOC was determined. The data are presented as the mean ± SD (n = 3 per group), and significance relative to the control group was determined using Duncan’s multiple-range test; *P < 0.05 and ***P < 0.001.

Toll-like receptor 4 (TLR4), in the form of TLR4/MD-2 complexes, is an important plant polysaccharide receptor^13^, and stimulation of TLR4 activates the myeloid differentiation primary response 88 (MyD88)-dependent pathway. We examined the protein levels of TLR4, MyD88, IRAK1 and TRAF6 in cells with oxidative stress that were treated with 20 μg/mL APS for different times by Western blotting assay. Our data showed the protein expression of TLR4 (*P* < 0.001) as well as MyD88, IRAK1 and TRAF6 (*P* < 0.05) was enhanced after 6 h of incubation, as shown in Fig. 3. However, nuclear factor-κB (NF-κB) was decreased after incubation with APS for 3 h. After incubation with APS for 9 h, expression of NF-κB was decreased (*P* < 0.01). Overexpression of RIG-I can promote inflammatory factors in cells. Figure 3F shows that RIG-I protein expression was reduced after incubation with APS (*P* < 0.001). In addition, RIG-I protein expression in the group incubated with 20 μg/mL APS for 6 h was lower than that in the other groups. To further confirm the role of TLR4 in the antioxidant effects of APS, we assessed whether TLR4 inhibitors affected the T-AOC in MEFs. Figure 3G shows that blocking the TLR4 receptor apparently reduced the T-AOC (*P* < 0.01).

### APS upregulated expression of MAPK and contributed to Nrf2 nuclear localization

**Figure 4:**
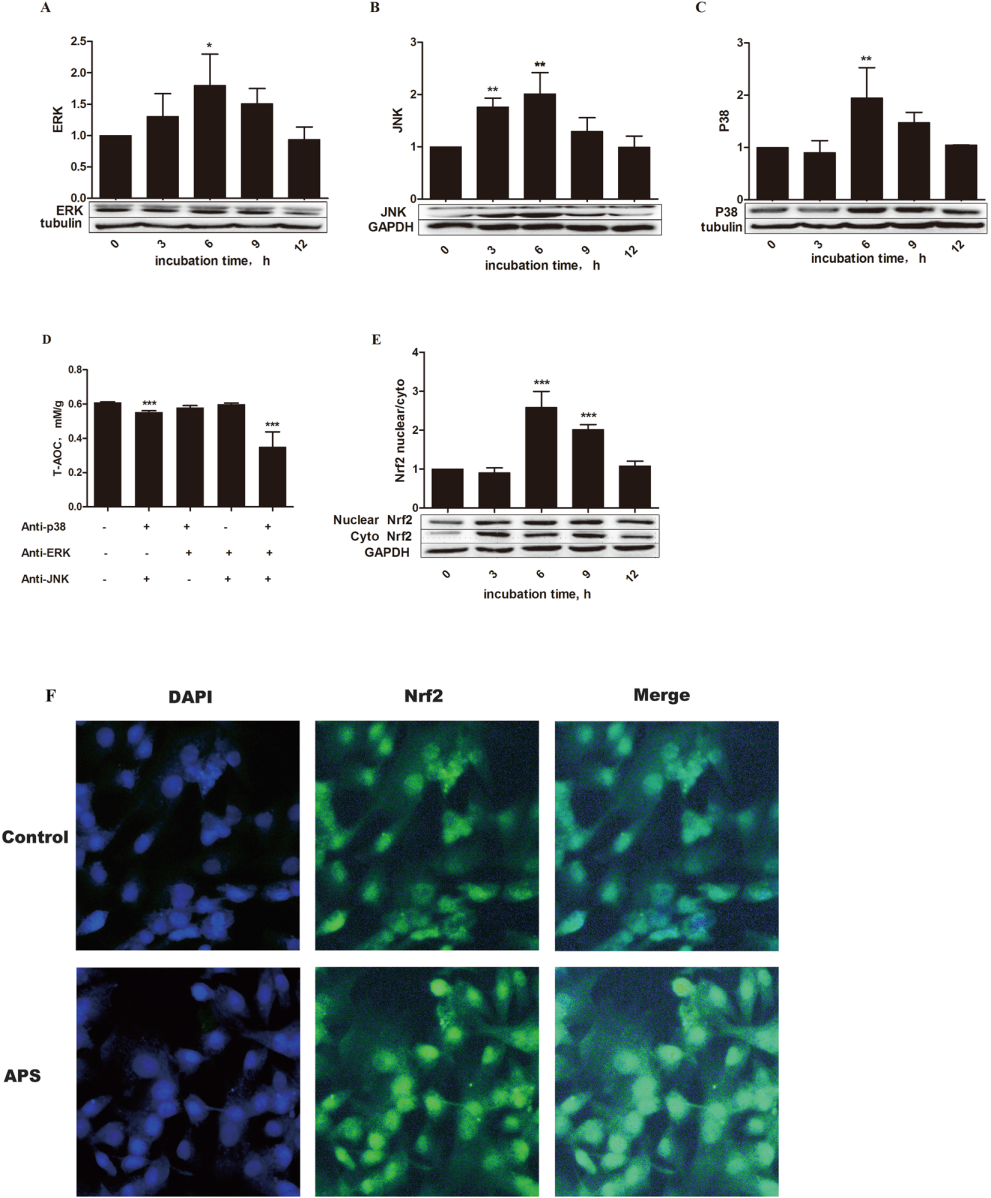
Effects of APS on expression of key proteins in the MAPK signaling pathway and on the nuclear translocation of Nrf2 in H_2_O_2_-pretreated MEFs. (**A**–**C**) After stimulation with H_2_O_2_ for 12 h, cells were incubated with 20 µg/mL APS for 0, 3, 6, 9, and 12 h, and expression of key elements was then assessed by Western blot analysis. The band intensities were analyzed using image analysis software. The values represent the mean ± SD (n = 3 per group). (**D**) After stimulation with 250 µM H_2_O_2_ for 12 h, MEFs were preincubated with inhibitors of p38 (SB203580, 20 μM), JNK (SP600125, 20 μM) and ERK (PD98059, 20 μM) for 1 h and then treated with 20 μg/mL APS for 6 h. Then, the T-AOC was determined at 405 nm using a microplate reader (Thermo Multiskan MK3, Thermo Fisher Scientific Inc., USA). The data are presented as the mean ± SD (n = 5 per group). Expression of Nrf2 was analyzed by Western blotting (**E**), and the ratio of nuclear Nrf2 to cytosolic Nrf2 is shown. The data are presented as the mean ± SD (n = 3 per group). (**F**) Immunohistochemical analysis was performed with an InCell Analyzer 2000 confocal microscope. The blue and green fluorescence indicate the localization of nucleus (DAPI) and Nrf2, respectively. The scale bars represent 100 µm. The significance relative to the control group was determined using Duncan’s multiple-range test; *P < 0.05, **P < 0.001, and ***P < 0.001.

Activation of the MyD88-dependent pathway results in rapid phosphorylation of mitogen-activated protein kinases (MAPKs) and NF-κB signaling. Figure 4 shows that the p38, ERK and JNK signaling pathways were activated by APS. In model cells incubated with 20 μg/mL APS, the protein levels of ERK (*P* < 0.05) and JNK and p38 (*P* < 0.01) were significantly increased compared to those in control cells. We further investigated whether MAPK pathways participate in the antioxidant effect of APS in MEFs. As shown in Fig. 4D, the T-AOC was decreased in cells treated with p38 and JNK inhibitors (anti-p38 and anti-JNK, respectively), APS and MAPK pathway inhibitors (*P* < 0.001) compared with that in control cells. However, this result was not observed in anti-ERK/anti-JNK-treated cells or anti-ERK/anti-p38-treated cells, suggesting that APS may regulate antioxidant activity via the p38 and JNK pathways.

Nuclear factor erythroid 2-related factor 2 (Nrf2) regulates the T-AOC via translocation between the nucleus and cytoplasm in response to various stress stimuli^24^. To further investigate whether APS can regulate Nrf2 localization, nuclear and cytoplasmic proteins were extracted to measure Nrf2. To visualize the results, we determined the displacement of Nrf2 by immunofluorescence. Nuclear translocation of the Nrf2 protein is essential for the protein to transactivate downstream genes, and phosphorylation of Nrf2 moderately enhances its nuclear accumulation^25^. The ratio of nuclear Nrf2 to cytosolic Nrf2 was increased (*P* < 0.001) in the APS groups, suggesting that the transcriptional activity of Nrf2 was increased. The fluorescent area and the fluorescence intensity of Nrf2 in the experimental groups were greater than those in the control group (Fig. 4). These results indicated that the antioxidative function of APS in cells was mediated by activation of the Nrf2 signaling pathway.

## Discussion

**Figure 5:**
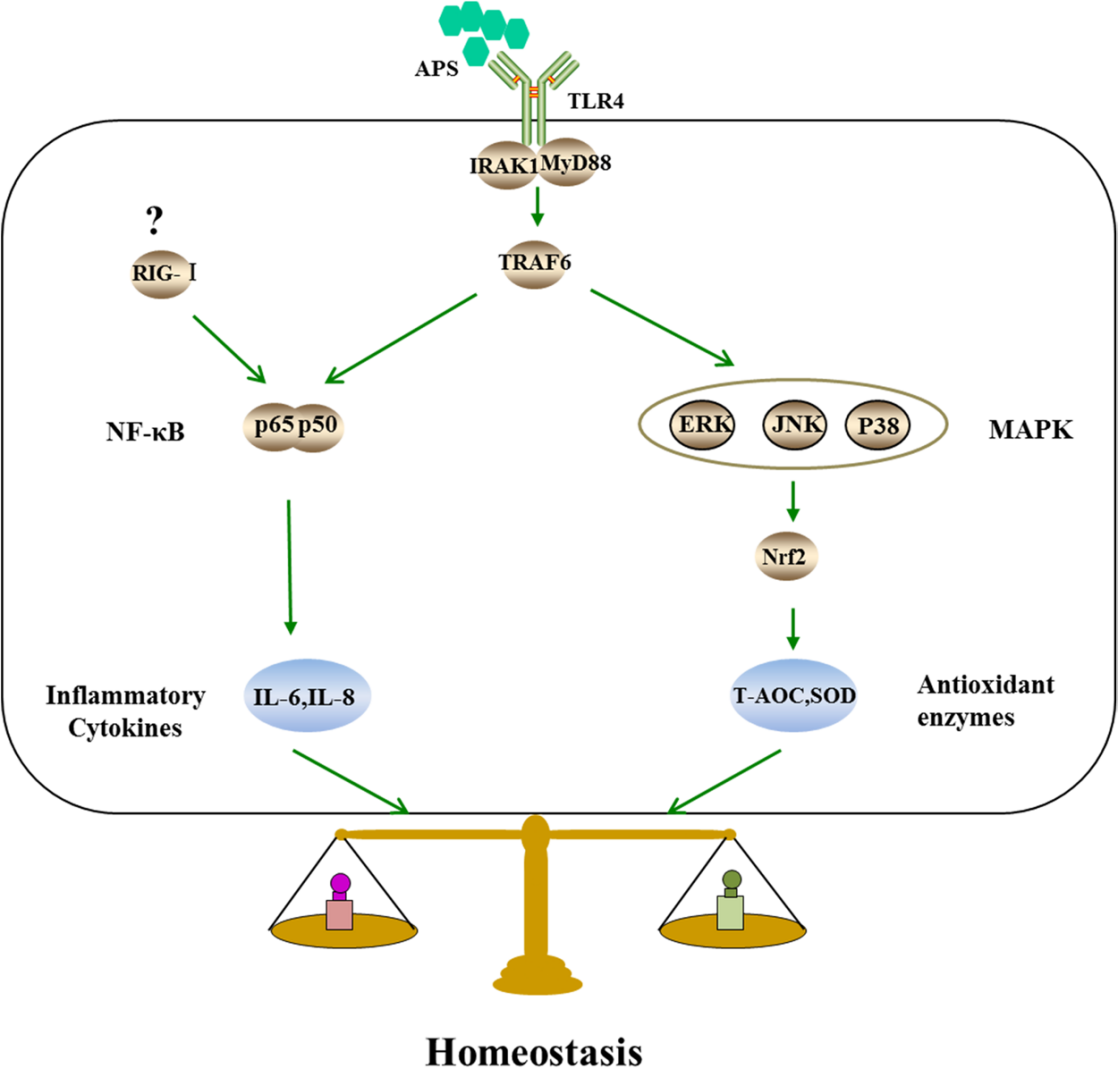
Speculated antioxidant related signaling pathways induced by APS in MEFs. (1) Because of the similarities in structure between APS and lipopolysaccharide (LPS), we speculate that TLR4 is likely the primary receptor of APS and that the subsequent signals are transmitted via MyD88, IRAK1 and TRAF6. (2) The two intracellular signaling pathways induced by APS may be (I) the MAPK signaling pathway including p38, JNK and ERK, which can induce the Nrf2 signaling pathway, and (II) the classical nuclear factor-κB (NF-κB) signaling pathway.

The first objective of this study was to characterize the anti-inflammatory function of APS, a polysaccharide isolated from alfalfa. TLR4, the first reported mammalian Toll-like receptor, is located on the cell surface, and upon activation, TLR4 interacts with myeloid differentiation primary response 88 (MyD88) protein adaptor molecules. The TLR4-MyD88 association further activates interleukin (IL) receptor-associated kinase (IRAK1) and tumor necrosis factor (TNF) receptor-associated factor 6 (TRAF6), which promote the transcription and expression of inflammatory factors^26^. Evidence has shown that TLR4 is an important receptor that associates with plant polysaccharides^27^. Therefore, we speculate that APS may activate the TLR4 receptor and then activate downstream elements. Our results strongly suggested that the proteins TLR4, MyD88, IRAK1 and TRAF6 were upregulated by APS. These results are in accordance with reports that *Reishi* polysaccharide extracts induce cytokine expression by activating TLR4^28^ and that *Polygonatum sibiricum* polysaccharide extracts activate TLR4 and upregulate expression of MyD88, IRAK1 and TRAF6 (Fig. 5)^29^. Unlike membrane-bound TLR4, RIG-I resides in the cytoplasm and recruits specific intracellular adaptor proteins to active NF-κB (Fig. 5). RIG-I can contact viral RNA to lead to the transcription of inflammatory cytokines^30^. Here, we propose, for the first time, that APS might suppress the expression of RIG-I. Although studies on the relationship between polysaccharides and RIG-I are limited, the present findings may provide a new foundation for exploring the mechanisms of APS and RIG-I. Furthermore, NF-κB binds to the inhibitory-type IκB protein in the cytoplasm. When oxidative damage occurs, IκB is phosphorylated and degraded. After being released, NF-κB translocates to the nucleus and binds to the nucleotide sequence of the κB domain to regulate the transcription of a variety of inflammatory cytokines. In the current study, we found that expression of NF-κB was suppressed by APS in H_2_O_2_-induced MEFs. This result is in accordance with the anti-inflammatory effects of apigenin^31^. These results, demonstrated that NF-κB is regulated oppositely by TLR4 and RIG-I. In addition, the effects of NF-κB were similar to those of RIG-I. Unfortunately, we could not determine how APS regulated the expression of RIG-I and NF-κB. Our data also showed that the T-AOC could be attenuated by a TLR4 inhibitor. This result is similar to the effect of oxyresveratrol^32^, which suggests that APS protects MEFs against H_2_O_2_-induced oxidative stress by enhancing the activity of TLR4.

Another objective of this study was to characterize the antioxidant function of APS. Increasing evidence suggests that many phytochemicals activate pathways that prevent or reverse oxidative injury^33^, and recent research has shown that the NF-κB and MAPK pathways are mediated by TLR4 dimerization in the cellular membrane^29,34^. MAPKs are a family of highly related kinases of which three major subfamilies are known: ERK, JNK, and p38. Although ERK mainly mediates the cellular response to hormones and growth factors, JNK and p38 are primarily activated by stress-related stimuli^35^. MAPK pathways are known to have important roles in regulating cellular responses to oxidative stress^36^. In fact, Nrf2 can serve as a downstream effector of MAPK to coordinate the mammalian defense system against oxidative stress^37^. Nrf2 has been demonstrated to be a critical transcription factor that binds to the antioxidant response element (ARE) in the promoter region of a number of genes, encoding for phase I and phase II antioxidative enzymes and cytoprotective proteins. Under normal conditions, Nrf2 is suppressed by Keap1 (Kelch-like ECH-associated protein-1). However, when this signaling pathway is activated, Nrf2 uncouples from Keap1, translocates to the nucleus, and binds to the ARE to regulate the transcription of phase II antioxidative enzymes. The MAPK signaling system responds to diverse stimuli, including oxidative stress, and has been implicated in Nrf2 induction in many previous reports (Fig. 5)^38^. Furthermore, *Ginkgo biloba* extract (EGB) can regulate MAPK expression in mouse C2C12 myoblasts^39^. This result is similar to our finding that APS can activate MAPK in MEFs. In addition, in this study, when MAPK signaling was blocked in MEFs by preincubation with an ERK-specific neutralizing antibody prior to APS addition, the T-AOC was significantly reduced. However, when members of the MAPK signaling pathways were not blocked, particularly p38 and JNK, the T-AOC was not significantly different from that of the control cells. These results demonstrated that APS regulates the antioxidative function of MEFs primarily via the p38 and JNK signaling pathways. We also discovered that APS significantly increased the level of Nrf2 in MEFs and promoted Nrf2 nuclear translocation. These results suggest that APS can protect cells against H_2_O_2_-induced oxidative stress by activating the MAPK/Nrf2 signaling pathway, which is in line with previous reports^40,41^.

Oxidative stress, which can cause various types of damage, such as senescence, necrosis, apoptosis, and inflammation^3,42,43^, is caused by a decrease in antioxidant defense and an increase in peroxide production. It has been reported that cells are dependent on antioxidant enzyme overexpression to resist oxidative stress^44^. SOD is the major antioxidant enzyme and the primary defense system against the ROS generated during oxidative stress. This enzyme catalyzes the breakdown of superoxide anion into oxygen and hydrogen peroxide^45,46^, and several studies have shown that elevated expression of SOD results in enhanced stress tolerance and increased life-span^47^. The T-AOC reflects the nonenzymatic antioxidant capacity against ROS and indicates oxidative stress or increased susceptibility to oxidative damage. It has been reported that cytochemically detectable β-galactosidase (β-gal) at pH 6.0 increases in senescence, and this protein has also been regarded as a marker of cellular senescence *in vivo* and *in vitro*^48^. In addition, evidence suggests that acute oxidative stress can shift cells into a state of senescence^49^. At the beginning of the 1990s, one study discovered that aging fibroblasts displayed a severe inflammatory reaction^50^. In addition, expression of IL-6 and IL-8 has been reported to play a role in oxidative stress-induced inflammation^51,52^, and in some cells, elevated IL-6 and IL-8 expression results in oxidative stress. This positive feedback loop may be important in the commitment of cells to an inflammatory response. Furthermore, senescence is associated with increased levels of IL-6 and IL-8, further supporting a key role for NF-κB in RIG-I-induced expression of IL-6 and IL-8 (Fig. 5)^21,53^. As detailed in Figs 1 and 2, we first constructed an oxidative stress cell model. Incubation of cells with 50 µM H_2_O_2_ reduced the T-AOC compared with control cells, but the concentration of IL-8 showed the opposite tendency. These results suggest that cells can resist weak oxidative stress. However, at higher H_2_O_2_ concentrations, the T-AOC and IL-6 and IL-8 levels were increased. This phenomenon also indicated that the balance between antioxidant and oxidant activity was destroyed^1^. Moreover, with the development of oxidative stress, RIG-I protein expression was increased, and the cells entered a state of senescence. However, we found that incubation with 20 µg/mL APS increased cell viability and reduced secretion of IL-6 and IL-8. These results are in line with those in reports on *Astragalus* polysaccharide, which increases cell proliferation and alleviates inflammation^54^. Another study has shown that plant extracts increase antioxidant enzyme activity in V79–4 cells^55^. Our results similarly showed that APS improved T-AOC and SOD activity against H_2_O_2_-induced oxidative damage.

In recent years, phytochemicals have been proposed to generally protect against oxidative stress^39,56–58^. In summary, we demonstrated that 20 μg/mL APS exhibited strong protective activity against H_2_O_2_-induced oxidative stress in MEFs through MAPK/Nrf2 and NF-κB pathways. This study provides new insight into the mechanisms underlying the protective effects of APS against oxidative stress. However, it is not clear how APS regulates RIG-I, which thus provides a new direction for research exploring the functions of APS. Although there are no specific standards for APS dosing or for the extraction process, the present results support the possibility that APS might be a good candidate compound to activate MAPK/Nrf2 and NF-κB signaling and thus treat oxidative damage.

## Conclusion

Understandably, there is tremendous public interest in the development of antioxidant therapies. If one accepts the evidence that APS plays a significant role in protecting against oxidative stress, strategies aimed at either reducing the oxidative burden or boosting host defense mechanisms involved in coping with the damage are likely to be considered. Two key issues will then arise. First, can effective therapeutics be designed to combat oxidative damage? Second, will these approaches be effective in slowing or preventing disease associated with oxidative stress?

This paper may provide the best answers to these questions. H_2_O_2_-induced oxidative stress induced MEF senescence, inflammation, and other injury. However, APS was able to alleviate oxidative damage. First, APS reduced expression of the RIG-I protein. The decrease in RIG-I suppressed NF-κB and then reduced secretion of IL-6 and IL-8, which can exacerbate oxidative damage. In addition, APS activated MAPK signaling, promoting Nrf2-Keap1 separation and, thus enabling the transcription of phase II antioxidative enzymes, such as SOD and T-AOC, which increase the capacity to resist oxidative stress.

## Materials and Methods

### MEF culture

This study, including the use of animals, was carried out in accordance with the recommendations of the Guide for the Care and Use of Laboratory Animals of Shandong Agricultural University Animal Nutrition Research Institute. All experimental protocols were approved by the Committee on the Ethics of Shandong Association of Animal Science and Veterinary Medicine.

Kunming mice were purchased from Taibang Biological Products (Co. Ltd. Tai’an, China). MEFs were isolated from 13 to 15-day-old embryos. In brief, pregnant mice were sacrificed and sterilized with medical alcohol (75%) for 2 min. Subsequently, embryos were isolated from the surrounding tissue. After removing the head, limbs and viscera, the residual tissues were cut into pieces and trypsinized. The cells were cultured in high glucose Dulbecco’s modified Eagle’s medium (DMEM, HyClone Laboratories, Inc., Logan, UT, USA) containing 5% fetal bovine serum (FBS, Solarbio, Beijing, China) and 1% penicillin-streptomycin (Solarbio, Beijing, China) at 5% CO_2_ in a 37 °C incubator. Nonadherent cells were removed by replacing the medium with fresh DMEM containing 5% FBS and 1% penicillin-streptomycin after one day of culture. The cells were trypsinized and passaged when they reached 80%-90% confluency.

### APS extraction and cell treatment

Alfalfa was planted on the farm of Shandong Agricultural University, and APS was extracted and purified in the laboratory of Shandong Agricultural University. The purity was 90.04%. APS was dissolved in culture medium and then filtered through sterilizing Millipore filters (pore size 0.22 μm) before use.

The current study comprised two major experiments. The first involved establishing the oxidative stress model and included three cell treatments: (1) Upon reaching 80–90% confluency, the cells were stimulated for 12 h with different concentrations of H_2_O_2_ (0, 50, 150, 250 and 500 µM) for the subsequent measurement of the T-AOC and IL-6 and IL-8 levels, which were used to determine the optimal concentration of H_2_O_2_ to construct the oxidative stress model (Figs 1A–C). (2) Cells were stimulated for 0, 6, 12, or 24 h with 250 µM H_2_O_2_. These cells expressed various related proteins, which were used to ensure the optimal incubation time with 250 µM H_2_O_2_ (Fig. 1D,E). (3) Cells were stimulated for 12 h with 250 µM H_2_O_2_ to determine whether the cells entered a state of senescence (Fig. 1F). The second experiment was performed to explore the antioxidant functions of APS on H_2_O_2_-induced MEFs and again included three treatments. (1) Cells were stimulated with 250 µM H_2_O_2_ for 12 h and then incubated with freshly prepared culture medium containing APS at a final concentration of 0, 5, 10, 20 or 30 μg/mL for 6 h. This assay was used to test cell viability, T-AOC, and SOD activity as well as IL-6 and IL-8 levels to determine the optimal APS concentration (Fig. 2). (2) MEFs were induced with 250 µM H_2_O_2_ for 12 h. After removing the culture medium, the cells were incubated with 20 μg/mL APS for 0, 3, 6, 9 or 12 h. The objective was to determine the optimal APS incubation time by determining the expression of related proteins (Figs 3 and 4) (3) Cells were stimulated with 250 µM H_2_O_2_ for 12 h, precultured with TLR4 and MAPK inhibitors for 1 h and then incubated with 20 μg/mL APS for 6 h (Figs 3G and 4D).

### Measurement of cell homogenate biochemical parameters

The first objective was to explore the optimal concentration of H_2_O_2_ to induce the model. The second objective was to determine the most suitable APS incubation conditions. For the experimental cell suspensions, cells were harvested using cell scrapers with phosphate-buffered saline (PBS) at pH 7.4 and centrifuged for 10 min at 1000 × g. The supernatant was discarded, and the cells were then resuspended in PBS. SOD levels in the samples were measured according to the manufacturer’s instructions (A001-3, Jiancheng Institute of Biotechnology, Nanjing, China), and the results are expressed as U/mgprotein. T-AOC was evaluated using commercial kits (A015-2, Jiancheng Institute of Biotechnology, Nanjing, China). The final concentration of T-AOC is expressed as mM/g protein.

The total protein content in the cells was measured using a BCA Protein Assay Kit (Beyotime Institute of Biotechnology, Beijing, China).

### Determination of IL-6 and IL-8

MEFs were homogenized, and the levels of the proinflammatory cytokine IL-6 and the chemokine IL-8 were determined with specific ELISA kits according to the manufacturer’s instructions (Lengton, China). The concentrations of IL-6 and IL-8 were measured using a spectrophotometer (Jasco V-530, Japan Servo Co. Ltd., Japan) at 450 nm. Standard curves were constructed by using standard cytokines and the concentrations of the unknown samples were calculated from the standard curves.

### Senescence-associated β-galactosidase (SA-β-gal) staining

Cultured cells were washed in PBS (pH 7.4) and then stained using a Senescent Cells Staining Kit (C0602, Beyotime Institute of Biotechnology, Jiangsu, China) according to the manufacturer’s instructions. The positive SA-β-gal cells (blue staining) were counted under a microscope. The experiments were performed in triplicate and at least 100 cells were analyzed in each treatment.

### Quantitative real-time PCR assay

Total RNA was extracted from the MEFs with an RNA-pure Tissue and Cell Kit (CW0584S, Cwbiotech, China) according to the manufacturer’s instructions. Purified total RNA was reverse transcribed into single-strand cDNA, which was successively analyzed by real-time qPCR using SYBR Green PCR master mix (Roche, Welwyn Garden City, Hertfordshire). The primers were synthesized by Ruibiotech as follows: RIG-I (forward): 5′-GGCATTTCCGTGTTTCTT-3′, RIG-I (reverse): 5′-GGTGGGCTTGGGATAGTC-3′^21^; β-actin (forward): 5′-CACTGTGCCCATCTACGA-3′, β-actin (reverse): 5′-CAGGATTCCATACCCAAG-3′^59^. The conditions for amplification included one cycle at 94 °C for 5 min, followed by 40 cycles at 94 °C for 20 sec, 57 °C for 20 sec, and 70 °C for 20 sec. A standard curve was prepared using a serial dilution of a reference sample and was created in each real-time run to correct for possible variations in product amplification. Relative copy numbers were obtained from the standard curve values and were normalized to the values obtained for the internal control, β-actin. The fold changes in expression were the obtained by the 2^−ΔΔCT^ method.

### Western blot analysis

Western blot analysis was divided into three components. First, we assessed whether H_2_O_2_ affected RIG-I. Second, we determined the important elements in related signaling pathways (TLR4-IRAK1, MAPK and NF-κB pathways). Finally, we detected Nrf2 protein in the nucleus and cytoplasm. Total protein was extracted from the cells according to the manufacturer’s instructions (P0013J, Beyotime Institute of Biotechnology, Jiangsu, China), and a Nuclear and Cytoplasmic Protein Extraction Kit (Beyotime Institute of Biotechnology, Beijing, China) was also used. First, we used a BCA kit (Beyotime Institute of Biotechnology, Beijing, China) to determine the protein concentration. Then, 30 µg of total protein and 20 µg of cytosolic and nuclear extracts from MEFs were separated using sodium dodecyl sulfate-polyacrylamide gel electrophoresis (SDS-PAGE), blotted onto a nitrocellulose membrane, and blocked with blocking buffer (Beyotime Institute of Biotechnology, China) at room temperature for 2 h. The membranes were then probed with antibodies for proteins in the upstream signaling pathway, including TLR4, IRAK1, MyD88, and TRAF6; those in internal signaling pathways in the nucleus and cytoplasm, including JNK, p38, ERK, NF-κB p65, and Nrf2; and β-actin (all antibodies were diluted 1:1000 and were purchased from Abcam, UK) at 4 °C overnight. The next day, after three washes with Tris-buffered saline with Tween 20 (TBST), the membranes were incubated with horseradish peroxidase (HRP)-conjugated goat anti-rabbit or anti-mouse IgG (Beyotime Institute of Biotechnology, China) at 4 °C for 4 h. Finally, the membranes were visualized with developing solution from a BeyoECL Plus Kit (P0018, Beyotime Institute of Biotechnology, China). The band areas were analyzed with a gel imager system (Alpha Innotech, San Leandro, USA).

### MTT proliferation assay

The proliferation activity of purified MEFs was determined according to the manufacturer’s instructions (C0009, Beyotime Institute of Biotechnology, Jiangsu, China) and then was measured by an enzyme-labeling instrument at 570 nm. The relative viability of the treated cells was calculated by comparing their optical density (OD) values with those of the control group.

### Nrf2 immunofluorescence

To assess Nrf2 displacement, we fixed cells in 4% paraformaldehyde and permeabilized them in 0.1% Triton X-100. After fixation and permeabilization, the cells were washed twice with PBS containing 1% bovine serum albumin (BSA) and incubated with an anti-Nrf2 antibody (1:100) overnight at 4 °C. The next day the slides were washed with PBS three times for 5 min each and then incubated with a FITC-conjugated secondary antibody (1:100) for 1 h at 37 °C. The slides were then washed three times in PBS, counterstained with 4′,6-diamidino-2-phenylindole (DAPI) for 5 min, and rinsed with PBS. The cells were visualized using an InCell Analyzer 2000 confocal microscope.

### Inhibitor treatment

To clarify the roles of the signaling pathways in MEFs, the cells were pretreated with or without inhibitors. We regarded the T-AOC as an index of inhibitor function. To block the interaction between TLR4 and APS, cells were pretreated with a TLR4-specific neutralizing antibody (Imgenex, San Diego, CA) for 1 h at 37 °C before incubation with 20 μg/mL APS for 6 h. Subsequently, we tested the T-AOC of the control and experimental groups.

Natural active polysaccharides mainly act on cells that activate MAPKs/Nrf2 by directly improving the expression and capacity of antioxidants and suppressing NF-κB, which can reduce inflammation^60^. Thus, to determine which pathway was activated by APS, MEFs were treated with the optimum concentration of APS one hour after adding the NF-κB inhibitor (PDTC, 750 nM), the p38 inhibitor (SB203580, 20 µM), the ERK inhibitor (PD98059, 20 µM) and the JNK inhibitor (SP600125, 20 µM). After incubating for 6 h, the cells were collected, and the expression of T-AOC was analyzed.

### Statistical analysis

The data were subjected to one-way analysis of variance (ANOVA), and the results are presented as means ± standard deviations (n = 3). Means were compared using Duncan’s multiple-range tests with SAS 9.2 software (SAS Institute Inc., Cary, NC, USA). Statistical significance is represented as *P < 0.05, **P < 0.01, and ***P < 0.001.
